# Modeling Virus-Associated Central Nervous System Disease in Non-Human Primates

**DOI:** 10.3390/ijms26146886

**Published:** 2025-07-17

**Authors:** Krystal J. Vail, Brittany N. Macha, Linh Hellmers, Tracy Fischer

**Affiliations:** Division of Comparative Pathology, Tulane National Primate Research Center, Covington, LA 70433, USA; bmacha@tulane.edu (B.N.M.); lhellmers@tulane.edu (L.H.); tfischer1@tulane.edu (T.F.)

**Keywords:** HIV, brain, inflammation, COVID, Zika virus, encephalitis, herpesvirus, West Nile virus

## Abstract

While viral pathogens are often subdivided into neurotropic and non-neurotropic categories, systemic inflammation caused by non-neurotropic viruses still possesses the ability to alter the central nervous system (CNS). Studies of CNS disease induced by viral infection, whether neurotropic or not, are presented with a unique set of challenges. First, because brain biopsies are rarely necessary to diagnose viral-associated neurological disorders, antemortem tissue samples are not readily available for study and human pathological studies must rely on end-stage, postmortem evaluations. Second, in vitro models fail to fully capture the nuances of an intact immune system, necessitating the use of animal models to fully characterize pathogenesis and identify potential therapeutic approaches. Non-human primates (NHP) represent a particularly attractive animal model in that they overcome many of the limits posed by more distant species and most closely mirror human disease pathogenesis and susceptibility. Here, we review NHP infection models of viruses known to infect and/or replicate within cells of the CNS, including West Nile virus, the equine encephalitis viruses, Zika virus, and herpesviruses, as well as those known to alter the immune status of the brain in the absence of significant CNS penetrance, including human immunodeficiency virus (HIV) in the current era of combination antiretroviral therapy (cART) and the coronavirus of severe acute respiratory syndrome (SARS)-CoV−2. This review focuses on viruses with an established role in causing CNS disease, including encephalitis, meningitis, and myelitis and NHP models of viral infection that are directly translatable to the human condition through relevant routes of infection, comparable disease pathogenesis, and responses to therapeutic intervention.

## 1. Introduction

Both neurotropic and non-neurotropic viral pathogens are capable of inducing changes in the central nervous system (CNS). Neurotropic viruses, defined here as viruses that directly infect CNS cells, such as neurons or microglia, often incite local cytokine or chemokine production and recruitment of inflammatory cells. However, even in the absence of true neurotropism, many viral pathogens influence the CNS immune milieu through interactions with viral proteins and/or factors associated with the systemic host immune response that influences microglial, astrocyte, and neuron activity, as well as cells comprising the blood–brain barrier (BBB), such as endothelial cells. For example, systemic inflammation is associated with impaired BBB integrity and microglial activation even in the absence of viral infection [1].

There are several challenges to interrogating viral pathogen-induced CNS disease. First, because noninvasive diagnostic methods are effective, invasive brain biopsies in living patients are uncommon. As a result, human brain tissue is not generally available until after death and human studies are largely limited to clinical observations and postmortem studies. In vitro models are increasingly sophisticated and enable cell-specific studies, but do not recapitulate a complete immune system, thus requiring the use of animal models to more completely model viral pathogenesis. Mouse models are the most widely used in vivo model given their small size, economical cost to maintain, genetic manipulability, and short breeding and life span, and are instrumental in studies identifying and screening potential therapeutic candidates. Nonetheless, the translation of mouse models to human disease is limited by vast differences in genetics, life span, anatomy, physiology, and metabolic biology, such that many discoveries made in mice that are often engineered or inherently identical fail to fully recapitulate human disease. Rodents also lack important human immune receptors, have significant anatomic differences including brain structure, and fail to reproduce some clinical symptoms observed in human disease. Non-human primates (NHP), particularly Old-World species such as macaques, anatomically and physiologically closely resemble human brain structure, immune system maturity, and BBB composition. Thus, NHPs bridge a key gap in studies modeling human disease pathogenesis and susceptibility, particularly CNS disease and provide a crucial platform for evaluating how viruses penetrate the BBB and trigger neuroinflammation.

Here, we review NHP models that recapitulate relevant aspects of human disease with an emphasis on routes of infection, comparable disease pathogenesis, and response to therapeutic intervention. We focus on viruses known to cause CNS disease and neuroinflammation, including encephalitis, meningitis, myelitis, and CNS proinflammatory cytokine production (Table 1). These include directly neuropathic viruses known to infect and/or replicate within cells of the CNS, such as West Nile virus, the equine encephalitis viruses, Zika virus, and herpesviruses, as well as non-neurotrophic viral pathogens known to perturb the immune milieu of the brain in the absence of significant viral penetrance into the CNS compartment, including human immunodeficiency virus (HIV) in the context of effective pharmacological intervention and the coronavirus of severe acute respiratory syndrome (SARS)-CoV-2.

## 2. Flaviviruses

The genus Flavivirus of family *Flaviviridae* includes Zika virus (ZIKV), Dengue virus, West Nile virus (WNV), yellow fever virus, and Japanese encephalitis virus. Here, we focus on ZIKV and WNV.

### 2.1. Zika Virus

ZIKV is an enveloped, RNA arbovirus initially isolated from a rhesus macaque (RM, *Macaca mulatta*) in the 1950s [2]. Disease due to ZIKV infection in humans is most often associated with mild rash, fever, conjunctivitis, and arthritis or joint pain [3] (Supplementary Table S1). Aedes mosquitos, especially *Aedes aegypti* are the primary vector, but ZIKV is also transmissible sexually and vertically during pregnancy from mother to fetus [4,5]. Indeed, ZIKV’s rise in public health significance is chiefly due to links between maternal infection and fetal loss and microcephaly, as well as the development of Guillain-Barre syndrome [6]. The virus has a particular tropism for neuroprogenitor cells (Supplementary Table S1), but also infects glia, epithelial cells, and leukocytes; cellular tropism has been reviewed in [7,8]. Furthermore, ZIKV nonstructural proteins NS4A and NS4B disrupt protein kinase B (Akt)- mammalian target of rapamycin (mTOR) and Notch signaling pathways critical for proper fetal brain development, resulting in impaired glial maturation, fetal myelination, and neurogenesis [9,10].

### 2.2. NHP Models of Zika Virus

To date, NHP models of ZIKV have centralized around two common themes: disease pathogenesis in adult and fetal infections, and vaccine development. There is currently no effective vaccine licensed to prevent ZIKV infection, although numerous vaccine candidates have been tested (reviewed in [11]). Viremia peaks 2–5 days post ZIKV-infection and macaques develop neutralizing antibodies [12,13]. Adult macaques develop rash and transient fever, with neurotropic strains such as the ZIKV strain associated with the 2013 epidemic in Brazil (ZIKVBR; Brazil/ZKV2015) resulting in neuroinflammation and disruption of the BBB [14] (Supplementary Table S1).

Congenital Zika syndrome, due to vertical transfer from dam to fetus, results in a range of placental and fetal neurodevelopmental abnormalities. Disease severity is primarily influenced by fetal age at infection with the most severe outcomes occurring in early gestation, and neurotropism of ZIKV sub lineage [15,16,17,18]. However, further studies are needed to fully elucidate how the relationship between sublineage neurotropism and gestational stage of infection influences Congenital Zika syndrome outcomes. Experimental gestational ZIKV (PRVABC59) exposure at the time of implantation resulted in impaired blastocyst attachment, embryonic degeneration, and reduced embryo secretion of pregnancy hormones [19]. ZIKV (PRVABC59) infection within the first and second trimester caused premature rupture of fetal membranes, premature cervical ripening, and placental abnormalities and inflammation [20,21]. Fetal pigtail macaques (*Macaca nemestrina*) infected in the second or early third trimester showed disruption of fetal myelin attributed to impaired oligodendrocyte maturation [22]. Infants surviving to birth showed seizure activity and motor delay, and a single rhesus infant developed features of dilated cardiomyopathy [21]. RM and pigtail macaque fetal brain lesions included mineralization, gliosis, ependymal and neuroprogenitor cell degeneration and loss, and ventricular enlargement (ZIKV strains F2213025-2010, PRVABC59, and Brazil/ZKV2015; Supplementary Table S1) [16,21,23,24,25]. In several cases, fetal brain lesions were observed in the absence of maternal clinical disease [24]. Importantly, one limitation of the NHP model is that while neuropathic strains can induce microscopic CNS lesions and reductions in fetal brain size, ZIKV-induced microcephaly is not observed in macaques [16,21,26].

### 2.3. West Nile Virus

WNV is a mosquito-borne flavivirus of humans, birds, horses and related animals that causes fever, flu-like symptoms, and rarely, fatal meningoencephalitis (Supplementary Table S2). WNV initially replicates in dendritic cells in the skin near the bite site, then travels hematogenously to the CNS where it primarily infects brain and spinal cord neurons, and to a lesser extent astrocytes; microglia and macrophages are less permissive to infection [27,28]. Neuroinvasive disease occurs via hematogenous spread, or viral peripheral nerve retrograde and anterograde axonal transport [29].

### 2.4. NHP Models of West Nile Virus

Old-World NHP species are naturally susceptible to WNV. Seroconversion occurs within 2 weeks of infection and regional seroprevalence in outdoor colonies coincides with that of humans [30,31,32,33,34]. However, as in humans, NHP WNV infection is usually subclinical by adding a layer of complexity to the utility of NHPs as a model of WNV neurological disease [30,31,32,35,36]. Clinical disease in humans is most commonly seen in WNV infections of elderly individuals, which to a limited extent, is mirrored in NHPs [37]. While an aged Barbary macaque (*Macaca sylvanus*) naturally infected with WNV developed meningoencephalitis and neurological signs, attempts to experimentally induce clinical disease in aged rhesus or cynomolgus macaques (CM, *Macaca fascicularis*) have been unsuccessful [36,38]. In studies comparing WNV immune outcomes across different age cohorts in NHPs, T cell proliferation was delayed in aged macaques compared with younger adults, but infection remained subclinical across age groups [36]. Instead, neuroinvasive WNV disease has been achieved through intracerebral inoculation, resulting in fever and neurological signs variably progressing to death [39,40,41,42]. This model has great potential for therapeutic development.

## 3. Alphaviruses

The genus Alphavirus consists of 29 viral species, including three encephalitis-causing arboviruses: Western, Eastern, and Venezuelan equine encephalitis viruses [43]. The equine encephalitis viruses (EEV) produce a self-limiting fever in most humans and horses, but may progress to serious neurological manifestations, including seizures and tremors, in up to 4% of cases [44,45]. Histological lesions of human EEV consist of mononuclear inflammation, demyelination, intracerebral hemorrhage, and necrosis of the brain and occasionally spinal cord, retina, or optic nerve (Supplementary Table S3) [46,47,48,49,50,51,52]. The viruses infect neurons, astrocytes, microglia, and oligodendroglia (Supplementary Table S3) [46,53]. Historically, diagnosis has relied on brain biopsy, but thankfully, modern next-generation sequencing of serum and cerebrospinal fluid (CSF) is diagnostic [54].

Importantly, aerosolized EEV are highly infectious and can cause fatal encephalitis; this potential for use as a biological weapon renders them a major threat to public health and safety [43,55]. Of note, aerosol transmission is not a natural route, necessitating the use of animal models to evaluate the pathogenesis of and countermeasures against aerosol transmission.

### 3.1. Eastern Equine Encephalitis Virus

Eastern equine encephalitis virus (EEEV) is the most pathogenic of the EEV in humans and NHPs [44]. Aerosol exposure to EEEV resulted in fever, seizures, and ultimately death in CMs and common marmosets due to encephalitis (Supplementary Table S3) [43,56,57,58,59]. Of note, the neuropathogenicity of EEEV does not appear to be restricted by route of infection, as subcutaneous inoculation also resulted in clinical signs and death similar to aerosol exposure [44].

### 3.2. Venezuelan Equine Encephalitis Virus

Venezuelan equine encephalitis virus (VEEV) consists of 6 subtypes, I–IV, divided into epizootic or enzootic strains that naturally cycle between Culex mosquitos and rodents; humans are susceptible to both subtypes. VEEV does not cause mortality in macaques except through intracranial inoculation [45,55,60]. CMs challenged with aerosolized VEEV developed fever, viremia, and ataxia [60,61,62,63,64] (Supplementary Table S3). RM challenged subcutaneously with epizootic subtypes developed fever, anorexia, diarrhea, and depression while CMs remained subclinical; rhesus macaques developed subclinical infections with enzootic strains [44,65]. The discrepancy in aerosol and subcutaneous neuropathogenicity is thought to be due to viral entry into the olfactory system and migration to the cerebral cortex where it infects neurons and astrocytes; although, the immunohistochemistry to support this has not been demonstrated in human or NHP autopsy specimens [45,53,66,67].

### 3.3. Western Equine Encephalitis Virus

In contrast to EEEV and VEEV, NHP studies of Western equine encephalitis virus (WEEV) are limited. Challenge with aerosolized WEEV resulted in fever, anorexia, and tremors, with fatality in 3 of 12 CMs [68] (Supplementary Table S3). Microscopically, inflammation extended into the brain, meninges, and to a lesser degree in the spinal cord [68]. Subcutaneous exposure resulted in a subclinical infection [44].

## 4. Herpesviruses

Herpesviruses are double-stranded DNA viruses capable of lifelong persistence as latent infections [69]. *Herpesviridae* is further subclassified into the subfamilies *Alphaherpesvirinae*, which includes the human herpesviruses varicella zoster virus (VZV), human herpes simplex virus 1 (HSV-1), and HSV-2; *Betaherpesvirinae*, which includes human cytomegalovirus (HCMV), human herpesvirus (HHV)-6, and HHV-7; and *Gammaherpesvirinae,* which includes Epstein–Barr virus, and Kaposi’s sarcoma herpesvirus (also known as HHV8) [69,70]. Here, we will focus on NHP models for VZV, HSV-1, HSV-2, and HCMV.

### 4.1. Varicella-Zoster Virus

VZV is a neurotropic alphaherpesvirus that initially causes varicella (chickenpox), establishes latency, and may later reactivate to cause herpes zoster (shingles). Infection occurs by direct contact with varicella lesions or inhalation of virus-laden droplets followed by viral replication in the upper respiratory tract and dissemination to the skin resulting in the characteristic vesicular rash (Supplementary Table S4) [71]. Following resolution of varicella, VZV establishes latency in the neurons of the dorsal root sensory ganglia and later reactivates in one-third of cases as herpes zoster, a painful, debilitating disease most common in adults over 50 years of age [72]. Reactivation is associated with aging, stress, organ transplantation, or immunosuppression, but may also occur in immunocompetent individuals [73]. Importantly, while herpes zoster is primarily a disease affecting the peripheral nervous system, reactivation can lead to life-threatening complications, including pneumonia and neurological involvement [74,75]. VZV encephalitis or meningitis manifests clinically as confusion, headache, nausea, and ataxia [73,75], and detection of VZV DNA in the CSF is diagnostic [76,77,78,79]. Like other herpesviruses, intranuclear viral inclusion bodies are observed microscopically but may be more prevalent in skin lesions [80]. Neurological illness is often associated with cerebrovascular disease, vasculitis, and increased risk of stroke, although encephalitis without vasculopathy has been reported [80,81].

### 4.2. NHP Models of Varicella-Zoster Virus

VZV is relatively species-specific and attempts to inoculate animals with VZV have failed to consistently induce varicella rash or clinical disease [82]. Simian varicella virus (SVV, Cercopithecine Herpesvirus 9) is an alphaherpesvirus of Old-World macaques that shares 70–75% DNA homology with VZV and causes clinical disease analogous to human VZV including vesicular rash, pneumonia, and less commonly, ganglioneuritis [83] (Supplementary Table S4). A key feature of SVV as a model for VZV is its ability to cause latent infection and reactivate upon immunosuppression or stress. Experimental reactivation is induced by T lymphocyte depletion via neutralizing antibodies or whole body radiation, alone or in combination with the stress induced by transport or relocation; these methods are effective at inducing SVV reactivation and development of zoster rash [83,84].

Latency in both VZV and SVV is characterized by restricted viral transcription with some differences between the two species. Viral transcription in human VZV latency is reduced to two latency-associated transcripts: VLT, which is antisense to open reading frame (ORF) 61, and ORF63 [85]. Both ORF 61 and 63 are expressed in SVV latency; however, 10 additional transcripts were identified in latently infected sensory ganglia, albeit at significantly lower levels [86]. SVV ORF 61 appears to be dispensable for infection as RM infection with a recombinant SVV lacking ORF61 had similar disease severity and viral latency in sensory ganglia wild-type SVV [87]. However, SVV ORF61 appears to interfere with host antiviral interferon response and NF-kB signaling [87,88]. Both VZV and SVV ORF 63 are expressed during latent and acute infection and impair antiviral interferon response through IRF9 degradation [89]. Additionally, macaques have also been used in vaccine development and testing [90,91].

CNS complications of human VZV infection include meningitis, myelitis, encephalitis, or vasculitis, with the primary risk factors being compromised immune status and age. SVV CNS involvement is also rare and studies of CNS involvement in NHP models are limited, despite the immunosuppressive nature of experimental reactivation. One study found a proinflammatory cytokine response in the CSF during primary SVV infection and reactivation, with variable correlation with serum levels [92]. VZV reactivation is also associated with vascular lesions, including cerebral vasculitis and stroke [81]. Vasculitis involving the lung and skin has been reported in SVV [93]. Serum substance P was elevated in SVV, which may be associated with increased risk of stroke; however, the studies of SVV vascular and CNS outcomes are incomplete [94].

### 4.3. Herpes Simplex Viruses

The alpha herpesviruses of herpes simplex viruses are divided into two serotypes: HSV-1 and HSV-2, both of which are widely prevalent; two-thirds of the global population are infected with HSV-1. Clinically, HSVs cause orofacial lesions, encephalitis, and genital lesions, due to their ability to infect most cell types, including immune cells, neurons, and epithelial cells (Supplementary Table S4). Both serotypes are capable of causing similar lesions; however, HSV-1 is more prevalent and more commonly associated with orofacial lesions due to preferentially establishing latency in A5-positive neurons and reactivating in the trigeminal ganglia [95]. Conversely, HSV-2 preferentially establishes latency in KH10 positive neurons and reactivation in the lumbar-sacral ganglia, and is most frequently found in genital herpes [96]. Upon entry through the skin or mucosa, HSVs undergo local lytic replication in the inoculation site, followed by retrograde transport along sensory nerve axons to peripheral sensory ganglia where they establish latency [97]. During stress-induced reactivation, HSV exits the neuron through anterograde transport and infects local cells or neurons, resulting in superficial mucocutaneous or CNS lesions [97]. Of further public health significance, HSV-2 infection is associated with a 3-fold increased risk of acquiring HIV, lending critical value to the NHP coinfection model in treatment and prevention strategies [98].

### 4.4. NHP Models of Herpes Simplex Virus

While many NHP viral infection models center on Old-World NHP species, HSV research spans both Old and New World models. Macaques (Old World monkeys) are susceptible to HSV infection, but less so than New World species such as marmosets (*Callithrix*) and owl monkeys (*Aotus*). This reduced susceptibility is thought to be due to simian Tripartite motif 5α (TRIM5α), which reduced HSV-1 and -2 replication in fibroblasts from RM and African green monkeys (AGM, *Chlorocebus sabaeus*) [99].

RM HSV-1 studies typically focus on oral and genital infection pathogenesis and development and testing of multivalent vaccines, microbicides, therapeutics, and prevention technologies [100,101,102,103,104,105,106,107,108]. RMs inoculated with HSV-1 via lip scratches developed oral vesicular lesions within 3–5 days, which resolved within 2 weeks but recurred months later in some cases [109]. CNS lesions included inflammatory cell infiltrates in the spinal cord and brain, and HSV-1 antigen was detected in these sites [109]. HSV-induced mucosal breaches facilitate HIV infection; macaque co-infection studies have also demonstrated that HSV-1 inoculation in SHIV- or SIV-infected animals resulted in vaginal inflammatory cytokine production, intermittent shedding, oral lesions, and detection of virus in trigeminal ganglia [110,111].

The utility of RMs as a model for HSV-2 is somewhat controversial; findings from at least one study suggested RMs are refractory to HSV-2 [112]. Alternatively, RMs challenged with a cocktail of HSV-2 strains demonstrated limited viral shedding, latency and reactivation, and histological lesions; however, anti-HSV-2 antibodies were only detected in 30% of animals [113]. HSV-2 failed to replicate in primary rhesus fibroblasts [114].

In contrast with macaques, New World monkeys, particularly marmosets and owl monkeys, are exquisitely sensitive to HSV infection, and are key models of HSV-1 encephalitis [115,116,117]. Marmosets naturally or experimentally infected with HSV-1 developed anorexia, dyspnea, ataxia, vesicular stomatitis, and meningoencephalitis [115,118,119,120]. In contrast, capuchins (*Cebus apella)*, another New World primate species, exhibited HSV-2 vulvovaginal vesicular lesions in the absence of neurological symptoms [114].

### 4.5. Cytomegalovirus

The betaherpesvirus human cytomegalovirus (HCMV), also known as human herpesvirus 5, establishes latency after primary infection resulting in lifelong infection with the potential for reactivation. While infection in immunocompetent individuals is usually subclinical, HCMV is a common opportunistic pathogen of the immunocompromised resulting in viral dissemination and disease [121]. Furthermore, congenital HCMV infection causes fetal and neonatal disorders in 10% of cases, including neurodevelopmental delays, sensorineural hearing loss, retinitis, or microcephaly [122,123,124] (Supplementary Table S4). Diagnosis often relies on viral isolation from urine or saliva, and microscopic evaluation often reveals the characteristic “owl’s eye” inclusion bodies [121,123]. Because CMV infection in immunocompetent individuals typically results in nonfatal outcomes, animal models are key for understanding pathogenesis.

### 4.6. NHP Models of Cytomegalovirus

One major challenge in HCMV research is that cytomegaloviruses are highly species-specific [125,126]. Fortuitously, human and rhesus (Rh)CMV are genetically and structurally similar, and RhCMV recapitulates many features of human HCMV infection rendering it a useful experimental model for HCMV [127,128]. Still, a barrier remains, as the vast majority of macaques in primate centers and wild colonies are RhCMV seropositive by one year of age, and, as in HCMV, RhCMV generally results in subclinical infections [129,130]. Historically, seropositive rhesus only developed clinical disease with SIV-induced immunodeficiency, resulting in multisystemic lesions [131]. Neuroinflammation may be observed with initial infection or reactivation in immunosuppressed or stressed animals.

The similarities between human and NHP reproductive biology lend relevance to the congenital macaque model, as rhesus pregnancies are also divided into trimesters and demonstrate similar placental and fetal development [132]. However, natural intrauterine transmission in rhesus is thought to be uncommon given the high seroprevalence and natural infection in endemic colonies and the fact that congenital transmission occurs most commonly when primary CMV infection occurs during pregnancy [129]. Experimental models using seronegative RMs have been modestly successful [133]. In one study, fetal loss was only observed in an acutely infected (at 8 weeks gestation) rhesus in which CD4^+^ T cells had been depleted; an immunocompetent dam not treated with CD4^+^ T cell depleting antibodies did not transmit CMV to her fetus [134].

## 5. Human Immunodeficiency Virus

The human immunodeficiency virus (HIV) belongs to the enveloped lentivirus subgroup within the retrovirus family. Each viral particle, or virion, encapsulates two copies of positive-sense, single-stranded viral RNA. This genetic material undergoes reverse transcription within the host cell, producing double-stranded DNA that is integrated into the host genome, establishing a covert life-long presence that defines the insidious nature of HIV infection. The virus predominantly targets CD4^+^ cells of the immune system, including macrophages, dendritic cells, and most notably, CD4^+^ T cells. Infection may culminate in the development of acquired immunodeficiency syndrome (AIDS), characterized by the progressive reduction in CD4^+^ T cells that results in loss of cell-mediated immunity and renders infected individuals susceptible to typically rare opportunistic infections, malignant neoplasms, and neurological injury.

Immune impairment and progression to AIDS have been markedly curbed in recent times through the implementation of highly effective combination antiretroviral therapy (cART). This therapeutic approach efficiently suppresses viral replication, facilitating immune system restoration, and consequently leading to substantial enhancements in both the quality and duration of life for individuals living with HIV. Yet, this does not cure individuals of infection and long-lived latently infected cells persist and establish stable tissue reservoirs of virus that recrudesce with cART cessation. Additionally, individuals undergoing successful cART experience significant health complications, such as cardiovascular disease, metabolic syndrome, and HIV-associated neurocognitive disorders (HAND). As such, HIV research that employs models that faithfully reflect the complexities of HIV infection and cART aids in dissecting the underlying mechanisms that foster comorbidities. This research not only enhances clinical management but also uncovers opportunities for preventive measures and tailored interventions to mitigate the risk of these comorbidities among individuals living with HIV.

### NHP Models of HIV: Simian Immunodeficiency Virus

Asian-origin macaques, including RMs infected with simian immunodeficiency virus (SIV) are widely recognized as the “gold standard” in HIV research, owing to their high translatability to various aspects of human disease. Several critical factors contribute to this status. Firstly, RMs are naturally vulnerable to SIV infection, which shares noteworthy similarities with HIV in critical aspects such as cell tropism, molecular structure, and the viral life cycle. Both viruses predominantly target CD4^+^ T cells, thereby causing immune dysfunction and eventually progressing to AIDS. This allows for in-depth exploration of viral-immune system interactions that closely emulate the human condition.

Another point of convergence between SIV and HIV is the molecular homology between the two viruses, which holds significant implications for the effectiveness of cART in SIV-infected RMs. HIV-targeting antiretroviral drugs operate by inhibiting key viral proteins essential for various life cycle stages, including viral attachment, fusion, reverse transcription, integration, assembly, and budding. Due to their structural similarity, SIV proteins are also susceptible to these antiretroviral agents, enabling effective therapeutic interventions. Of equal importance is the alignment of drug resistance mechanisms between SIV and HIV. Investigations into the development of drug resistance in SIV can thereby yield actionable insights into corresponding strategies for managing or preventing resistance in HIV therapies. Furthermore, the compatibility of cART in SIV-infected RMs facilitates research into reservoir-targeting strategies, a central area of focus in current efforts to achieve a functional cure for HIV. Although several of these therapeutic strategies show promise, it is crucial to note that the majority remain in the experimental phase, with many being tested primarily in animal models like SIV-infected RMs.

Despite these many parallels, several notable differences exist between SIV infection in RMs and HIV infection in humans, particularly related to the anatomical distribution, cellular composition, and establishment of latent viral reservoirs. In HIV-infected humans, viral replication and latency are predominantly localized in secondary lymphoid tissues such as lymph nodes, spleen, and gut-associated lymphoid tissue (GALT), as well as in long-lived memory CD4^+^ T cell subsets. Tissue tropism in cART-treated SIV-infected rhesus macaques reflects important nuances in reservoir localization and persistence that are critical for modeling HIV pathogenesis and cure strategies. Following infection, SIV disseminates rapidly to multiple tissue compartments, including gut-associated lymphoid tissue (GALT), lymph nodes, spleen, and CNS. Even under effective cART, viral DNA and replication-competent virus persist in these tissues, forming stable reservoirs that are refractory to immune clearance and therapeutic suppression [135,136,137]. Among these, the gastrointestinal mucosa and lymphoid tissues remain major sites of latent infection, harboring infected CD4^+^ T cells, particularly within transitional and effector memory subsets, despite prolonged viral suppression.

Within the CNS, perivascular and meningeal macrophages represent the principal cellular reservoirs of SIV during suppressive cART [138]. These cells can harbor integrated viral DNA, even in the absence of detectable plasma viremia. Microglia, while susceptible to SIV infection, appear to be less consistently infected during cART, and their contribution to the persistent CNS reservoir may depend on factors such as timing of therapy initiation and the neurotropism of the viral strain used. Importantly, low-level viral RNA and chronic neuroinflammation can be detected in the CNS of cART-treated macaques, implicating this compartment as both a sanctuary site and a contributor to HAND-like pathology [139].

The choice of viral strain significantly influences tissue tropism and reservoir dynamics. SIVmac239, a neurovirulent, molecularly cloned strain, exhibits robust seeding of peripheral and CNS reservoirs and is frequently used in cure and pathogenesis studies due to its high replicative fitness and consistent induction of systemic and CNS infection. SIVmac251, a more genetically heterogeneous quasispecies, demonstrates broader mucosal transmissibility and sustained infection across lymphoid and non-lymphoid tissues, while also supporting CNS persistence during long-term cART [140]. While both strains permit rigorous evaluation of tissue reservoirs and therapeutic interventions targeting viral latency, differences in viral dynamics must be accounted for in experimental design and data interpretation.

In addition to the significant relevance of macaques in therapeutic development and cure strategies for HIV, RMs are invaluable models for many key aspects of HIV neuropathogenesis. Due to the responsiveness of SIV-infected RMs to cART, they provide a critical platform for investigating the mechanisms and dynamics of the entry, replication, and potential persistence of SIV/HIV in the brain in the context of current antiretroviral treatment [138,141,142]. Chronic neuroinflammation remains a hallmark of HIV neuropathogenesis, even in the current era of effective cART, and likely plays a major role in HAND development and progression [143,144,145,146]. SIV-infected, cART-treated RMs also demonstrate evidence of persistent neuroinflammation [147], allowing researchers to examine the inflammatory mechanisms in the brain that underly neuronal injury and cognitive dysfunction in the context of virus suppression and immune restoration, which cannot be fully investigated in living human subjects.

## 6. Coronaviruses of Severe Acute Respiratory Syndrome

The Betacoronavirus genus of the *Coronaviridae* family comprises several viruses that primarily cause respiratory disease. Notable among these include severe acute respiratory syndrome coronavirus (SARS-CoV), Middle East respiratory syndrome coronavirus, and most recently, SARS-CoV-2. Each of these viruses has been linked to pandemics in the last two decades. Our focus here is on SARS-CoV-2, the etiologic agent of coronavirus disease 2019 (COVID-19), which emerged in 2019. Coronaviruses are enveloped, positive-sense, single-stranded RNA viruses, containing several structural proteins associated with the viral envelope, a ribonucleoprotein complex comprising the viral genome and nucleocapsid protein (N), and a replicase complex that encodes nonstructural proteins [148,149,150]. Nonstructural proteins include proteases and RNA-dependent RNA polymerase (RdRp) that enable viral replication. Structural proteins, spike (S), membrane (M), and envelope (E) are anchored within and comprise the majority of the viral envelope.

Coronavirus S proteins are critical for infection by facilitating cell binding and fusion through interaction with host cell receptors, such as the angiotensin-converting enzyme-2 (ACE2) receptor required for the entry of SARS-CoV-2 into host cells [151]. SARS-CoV-2 transmission typically occurs through the inhalation of contaminated droplets. The viral S protein binds the host cell receptors ACE2 and serine protease transmembrane 2 (TMPRSS2), facilitating viral entry into the host cell cytoplasm. This entry allows the viral RNA genome to be translated into structural proteins and polyproteins, promoting viral replication and the release of new viral particles.

In humans, SARS-CoV-2 infection presents a spectrum of clinical symptoms, ranging from asymptomatic or mild infection to severe manifestations, such as respiratory distress and multiorgan failure. A major challenge in combating the COVID-19 pandemic has been the long-term and extrapulmonary effects of infection. These include long-COVID, respiratory disease, and notable neurological manifestations, including headache and neurocognitive impairment.

### NHP Models of SARS-CoV-2

NHPs, particularly the macaques, serve as ideal models for studying SARS-CoV-2 pathogenesis and vaccine development. They are naturally permissive to infection and accurately model clinical pathology and immunologic features of human disease [152]. Due to the high conservation of the ACE2 amino acid sequence between RMs, CMs, and humans, as well as similarities in immune responses, NHPs are crucial models for testing vaccine efficacy [153,154]. Like humans, SARS-CoV-2-infected NHPs exhibit transient viral replication in the airways, and often mild, self-limiting lung inflammation [152,155,156]; however, some study subjects develop more severe infection, characterized by respiratory distress, severe broncho-interstitial pneumonia, and, in extreme cases, death [157].

NHP models of SARS-CoV-2 infection also reflect the neurological complications of infection observed in humans. A study examining the CSF proteome post-infection in AGMs and RMs revealed an enrichment of CSF proteins associated with neuroinflammation, innate immunity, and hemostasis [158]. This is consistent with human studies, where CSF leukocytes exhibited an enhanced inflammatory signature [159]. Additionally, the brains of infected RMs and AGMs demonstrated marked neuroinflammation, microhemorrhages, and neuron degeneration and apoptosis [160], paralleling neuropathological changes reported in humans [161,162]. Notably, CNS injury and neuroinflammation were observed in animals at approximately 4 weeks post-infection and in animals that did not experience severe respiratory disease [160], suggesting ongoing neurological involvement after resolution of acute infection.

SARS-CoV-2 enters host cells via ACE2, which is expressed not only in the respiratory tract but also in various brain regions, including the olfactory bulb, hippocampus, hypothalamus, and brainstem. Variable ACE2 expression in these areas has been confirmed in both human and NHP brains [163,164], raising the possibility that SARS-CoV-2 may access the CNS through neural pathways such as the olfactory nerve. Indeed, viral RNA and protein have been detected in the olfactory bulb and associated cortices in both human postmortem tissue [165] and infected NHPs [166], supporting the hypothesis of transneuronal viral spread. These findings may explain common clinical manifestations such as anosmia and ageusia during acute infection [167]. Further, ACE2 expression in limbic and hypothalamic structures suggests that direct viral effects and neuroinflammation in these regions may contribute to neuropsychiatric symptoms reported during and after infection, including depression, anxiety, and cognitive impairment [168,169,170]. Importantly, infected NHPs exhibit robust transcriptional and histopathological alterations indicative of synaptic plasticity changes, neuroinflammation, and cerebrovascular injury, even in the absence of detectable virus [160,171], providing important mechanistic insight into neurocognitive disturbances and mood disorders that can persist long after viral clearance.

Together, these data underscore the utility of NHP models not only for characterizing the systemic and pulmonary manifestations of SARS-CoV-2 infection but also for elucidating potential routes for viral neuroinvasion and the impact of the virus on CNS function. The anatomical and functional parallels between NHP and human brains, including ACE2 distribution, make these models especially valuable for dissecting the neurobiological substrates of long COVID.

## 7. Discussion

Within the past few decades, events such as the Zika virus epidemic and the SARS-CoV-2 pandemic have highlighted the importance of appropriate research studies of viral pathogens. Animal models are critical for overcoming the challenges encountered in recapitulating human viral infections, particularly outcomes on the brain and the interaction between the central nervous system and the body. There are several limitations of NHP models and future directions of viral-associated CNS disease in NHP models we would like to highlight. The NHP model does not always fully mirror human disease, as seen with ZIKV microcephaly and HSV-2 infection. Others mirror human subclinical infection and require experimental immunosuppression to consistently achieve reactivation or clinical disease.

WNV encephalitis generally impacts elderly patients with co-morbidities, which likely exacerbate age-related immune suppression. While antiviral T-cell proliferative responses appeared to be delayed in aged macaques experimentally infected with WNV, aging alone was not sufficient to permit neuroinvasive disease. Natural WNV infection of an aged Barbary macaque resulted in encephalitis; this animal had an underlying co-morbidity (osteoarthritis), which may have contributed to a higher baseline inflammatory level, tipping the balance in favor of disease progression [38]. Further study is needed to define the interface between aging, specific co-morbidities, and progression to WNV encephalitis in NHP models.

One major caveat to the NHP model for VZV is that while neurological involvements, such as meningitis or stroke, are grave complications of VZV reactivation, CNS complications are not well described in NHP SVV infections. Additional studies are needed to characterize the pathological changes in the brain, vasculature, and CSF during acute SVV infection as well as during reactivation.

NHP models enable longitudinal studies of natural disease and probe the cellular and molecular outcomes during the progression of disease in an intact immune system, with more complete and thorough characterization of therapeutic efficacy or toxicity. For many viruses, NHP models represent a highly analogous model for human disease due to parallels in life span, metabolic and reproductive biology. Additionally, genetic similarities particularly with host-specific viruses and analogous pathogens render the NHP model invaluable.

## Figures and Tables

**Table 1:** NHP models of CNS-associated viral disease.

Virus	Neurotropism	Advantages of NHP Model	Disadvantages of NHP Model
Zika virus	Directly neurotropic	Recapitulates human clinical disease	Microcephaly not observed in RM
West Nile virus	Directly neurotropic	Macaques are naturally susceptible	Most infections are subclinical
Eastern equine encephalitis virus	Directly neurotropic	Aerosol infection model	Most infections are subclinical
Venezuelan equine encephalitis virus	Directly neurotropic	Recapitulates human disease symptoms	
Western equine encephalitis virus	Directly neurotropic	Aerosol infection model	Limited studies
Varicella -zoster virus	Directly neurotropic	Recapitulates human disease, including latency	Limited CNS disease
Herpes simplex virus-1	Directly neurotropic	Latency modeling	Limited CNS disease in macaques
Herpes simplex virus-2 (HSV-2)	Directly neurotropic	HIV co-infection models	Macaques are refractory to HSV-2
Cytomegalovirus (CMV)	Directly neurotropic	Structural and genetic similarities between HCMV and RhCMV	Infections often subclinical, limited seronegative colonies
HIV	Indirectly neuropathogenic	Virus homology with SIV, recapitulates human clinical disease progression	Limited to Asian-origin macaques
SARS-CoV-2	Indirectly neuropathogenic	Naturally susceptible and recapitulate human disease progression including neurological complications and long COVID-19	

Abbreviations: NHP, non-human primate; RM, rhesus macaque; CNS, central nervous system; HIV, human immunodeficiency virus; SIV, simian immunodeficiency virus; SARS-CoV-2, severe acute respiratory syndrome coronavirus; COVID-19, coronavirus disease 2019.

## Data Availability

No new data were created.

## Supplementary Materials

The supporting information can be downloaded at https://www.mdpi.com/article/10.3390/ijms26146886/s1.

## Abbreviations

The following abbreviations are used in this manuscript:

ACE-2	Angiotensin-Converting Enzyme-2
AGM	African green monkey
AIDS	Acquired immunodeficiency syndrome
cART	Combination antiretroviral therapy
CNS	Central nervous system
COVID-19	Coronavirus disease 2019
CM	Cynomolgus macaque
EEEV	Eastern equine encephalitis virus
EEV	Equine encephalitis virus
HAND	HIV-associated neurocognitive disorders
HIV	Human immunodeficiency virus
HCMV	Human cytomegalovirus
HSV	Herpes simplex virus
NHP	Non-human primate
ORF	Open reading frame
RM	Rhesus macaque
RhCMV	Rhesus cytomegalovirus
SARS-CoV-2	Coronavirus of severe acute respiratory syndrome
SIV	Simian immunodeficiency virus
SVV	Simian varicella virus
VEEV	Venezuelan equine encephalitis virus
VZV	Varicella zoster virus
WEEE	Western equine encephalitis virus
WNV	West Nile virus
ZIKV	Zika virus
