# Supplementary material for: Modeling Virus-Associated Central Nervous System Disease in Non-Human Primates
Source: Int J Mol Sci. 2025 Jul 17;26(14):6886. doi: 10.3390/ijms26146886 (PMC12295480; doi:10.3390/ijms26146886)
Supplement: Supplementary file 1 [file ijms-26-06886-s001.zip › ijms-3714270-supplementary.pdf]

Supplemental Table S1: Zika virus (ZKV)

Species	Clinical Signs	Gross Lesions	Microscopic Lesions	Route of infection	Cells infected
Human	Rash, fever, arthritis or joint pain, conjunctivitis, headache [3]<br><br>Congenital infection: intrauterine growth restriction, fetal loss, sensorineural hearing loss and blindness [7]	Congenital ZKV: microcephaly, calcification, hypoplasia, arthrygroposis, ventricular dilation, micropthalmia; pulmonary hypoplasia [7]	Placental inflammation (villitis, fibrosis, chorionic vasculitis, deciduitis); cerebral and cerebellar hypoplasia, dysplasia, and degeneration	Mosquito, sexual, congenital []. Detectable in urine and saliva. [3,4,5]	Neuroprogenitor cells, neurons, cranial neural crest cells, astrocytes, microglia, glioblastoma cell line, olfactory epithelial cells, fibroblasts, trophoblast stem cells, lymphocytes and monocytes [7,8]
Pigtail macaque (*Macaca nemestrina*)	Fetal growth deceleration [24], Fetal loss [26];	Rash, rectal bleeding [23]	Fetal ependymal injury, gliosis, cerebral white matter hypoplasia, axonal injury, ventricular enlargement, reduction in neuroprogenitor cells [24,23]	Maternal subcutaneous challenge	Neuroprogenitor cells
Rhesus macaque (*Macaca mulatta*)– pregnant adult/ fetus	Abortion, in utero death, adult conjunctivitis, rash, fever, premature rupture of fetal membranes, premature cervical ripening, seizure activity, motor delay; Adult fever [14.16,21,25]	Maternal bloody vaginal discharge. Fetal/infant focal lissencephaly, decreased head circumference, fetal cardiomegaly, meningeal hemorrhage [21,25]	Fetal/infant CNS lesions: Periventricular calcification, gliosis, neural precursor cell apoptosis, ependymal and Purkinje cell loss/degeneration; hemorrhage, spinal cord meningitis, myelitis, and degeneration, [16,21]<br><br>Other lesions: impaired blastocyst attachment, embryonic degeneration, necrosuppurative placentitis, decidualitis, spiral artery leukoclastic vasculitis, fetal pneumonia, dermatitis, fetal cystitis, testicular hemorrhage, fetal retinitis [15,16,19,20, 25]	Maternal subcutaneous challenge	Neuroprogenitor cells
Olive baboon (*Papio Anubis*) – adult and fetus	Fetal death, adult (male and female) rash, conjunctivitis [17]	Rash [17]	Fetal neuroinflammation, astrogliosis, microgliosis [17]	Subcutaneous	

Supplemental Table S2: West Nile virus

Species	Clinical Signs	Gross Lesions	Microscopic Lesions	Route of infection	Cells infected
Human	Fever, flaccid limb paralysis/ muscle weakness [37]	Meningitis, encephalitis, acute flaccid paralysis	Mononuclear encephalitis or meningoencephalitis[37]	Mosquito.	Neurons, astrocytes, monocyte/ macrophage, retrograde and anterograde axonal transport and transneuronal spread [28,29]
Rhesus macaque (Macaca mulatta), cynomolgus macaque (*Macaca fascicularis*), hamadryas baboon (*Papio hamadryas*)	Subclinical; Fever, lethargy, ataxia, convulsions, death [31,33,35,36,40,42]	None	None or encephalitis, gliosis, neuronal necrosis, and loss of cerebellar Purkinje cells [40,42]	Spontaneous (mosquito presumed)<br><br>Intrathalamic inoculation	
Barbary ape (*Macaca sylvanus*)	Ataxia, ptyalism, nystagmus, shaking, and apathy [38]		Nonsuppurative meningoencephalitis with gliosis, glial nodules, and cuffs of mononuclear inflammatory cells around vessels [38]	Spontaneous (mosquito presumed)	

Supplemental Table S3: Alphaviruses: equine encephalitis viruses (EEV)

Eastern EEV	Clinical Signs	Gross Lesions	Microscopic Lesions	Route of infection	Cells infected
Human	Fever, headache, seizures, muscle rigidity or weakness [46]	Cerebral edema, meningitis, vascular congestion [46]	Meningoencephalitis, demyelination; retinitis [46,52]	Mosquito	Neurons, astrocytes, oligodendroglia. [46,66]
Cynomolgus macaque (*Macaca fascicularis*),	Fever, increased respiratory and heart rate, elevated blood pressure, cardiac abnormalities, seizures, tremors, anorexia, nystagmus, ataxia, death [-57,58,]	No gross lesions [44]	Mononuclear meningoencephalitis with neuronal necrosis and hemorrhage; gliosis, vasculitis [44,56]	Aerosol; subcutaneous	
Common marmoset (*Callithrix jacchus*)	Subclinical, anorexia, fever, inactivity/depression, abnormal blinking, death [59]	No gross lesions [59]	Meningoencephalitis, neuronal necrosis, hemorrhage, retinitis [59]	Aerosol/ Intranasal	Neurons

Venezuelan EEV	Clinical Signs	Gross Lesions	Microscopic Lesions	Route of infection	Cells infected
Human	Fever, chills, headache, backpain, malaise, myalgia, anorexia, nausea, sore throat, lymphopenia	Pharyngeal erythema, pharyngeal edema lymphadenopathy	Inflammation in the brain, lymph nodes, spleen, liver, lungs, and gastrointestinal tract	Mosquito, laboratory acquired (percutaneous or aerosol)	Neurons, olfactory neuroepithelium, astrocytes, Langerhans/ dendritic cells, lymphocytes [53,66]
Cynomolgus macaque	Fever or hypothermia, viremia, lymphopenia; anorexia, lethargy, ataxia [60-64]	No gross lesions	Neuronal necrosis and gliosis in cerebrum; meningitis [44]	Aerosol; subcutaneous	
Rhesus macaque (*Macaca mulatta*)	Subclinical, fever, anorexia, diarrhea, depression [44,65]			Aerosol, intranasal	

Western EEV	Clinical Signs	Gross Lesions	Microscopic Lesions	Route of infection	Cells infected
Human	Subclinical; fever, seizures, diarrhea [48]	Cerebral edema with sclerosis and nodules [48]	Cerebral gliosis, lymphohistiocytic encephalitis [48]	Mosquito	
Cynomolgus macaque	Fever, chills, headache, backpain, malaise, nausea/anorexia, tremors, transient leukocytosis, neutrophilia, death [68]	Meningeal congestion [68]	Encephalitis, meningitis, necrosis, hemorrhage, demyelination, , glial nodules [68]	Aerosol	Neurons, microglia, cerebellar Purkinje cells [68]

Supplemental Table S4: Herpesviruses

VZV/SVV	Clinical Signs	Gross Lesions	Microscopic Lesions	Route of infection	Cells infected
Human	Fever, nausea and vomiting, meningoencephalitis [80]	Vesicular rash; cerebral edema; interstitial pneumonia	Encephalitis (neutrophilic and histiocytic); vesicular dermatitis; multinucleated cells with intranuclear viral inclusion bodies[80]	Aerosol, direct contact with vesicular fluid	Epithelial cells, ganglionic neuronal cells, astrocytes, lymphocytes [80,81]
Rhesus macaque (*Macaca mulatta*)	Fever, lethargy, ataxia, viremia [94,83]	Vesicular rash,; disseminated necrosis (immunocompromised) [94,83]	Vesicular dermatitis with epithelial cell intranuclear inclusions and syncytia, pneumonia, adrenalitis with Cowdry A inclusion bodies []	Aerosol (intrabronchial, intratracheal)	Neurons, epithelial cells, macrophages, alveolar pneumocytes
Cynomolgus macaque (*Macaca fascicularis*)	Fever, dyspnea	Interstitial pneumonia and edema	Interstitial pneumonia with epithelial necrosis	Intratracheal	Epithelial cells, pneumocytes, lymphocytes, ganglia

HSV	Clinical Signs	Gross Lesions	Microscopic Lesions	Route of infection	Cells infected
Human HSV-1 and HSV-2	Hemiparesis, aphasia, seizures	Orofacial and genital lesions	Encephalitis	Skin/ mucosal of oropharynx or genitalia	Epithelial cells, neurons, fibroblasts, lymphocytes [69,97]
Rhesus macaque	HSV-1: Fever<br>HSV-2: Vaginal secretion, variable latent infection and reactivation [113]	HSV-1: Oral vesicular lesions [109]	HSV-1: Oral vesicular lesions [109]<br>HSV-2: Epithelial cell acantholysis, ballooning degeneration and intranuclear inclusion bodies [113]	Vaginal mucosa, oral mucosa [109,110,113]	HSV-1: Epithelial cells, neurons, astrocytes [109]<br>HSV-2: Epithelial cells, keratinocytes, neurons [113]
Marmosets Black tufted (*Callithrix penincillata*); common (*C. jacchus*); pigmy (*C. pygmaea*)[115,118,119]	HSV-1: Apathy, anorexia, dyspnea, ataxia, hypersalivation, [115,118,119,120]	HSV-1: Conjunctivitis, vesicular/ulcerative dermatitis, stomatitis, and glossitis [115,118,119]	HSV-1: Nonsuppurative meningoencephalitis with mononuclear perivascular cuffs and gliosis<br>epithelial intranuclear viral inclusion bodies, adrenalitis and hemorrhage [115,118,119,120].		HSV-1: Neurons, epithelial cells, neurons, peripheral nerves, monocytes, hepatocytes [119]

CMV	Clinical Signs	Gross Lesions	Microscopic Lesions	Route of infection	Cells infected
Human	Subclinical; prematurity, neuro-developmental delays, hearing loss, retinitis [86,87]	Low birth weight	Ventriculitis, asymmetric polyradiculopathy	Congenital	Epithelial, dendritic, endothelial hematopoietic progenitor, smooth muscle cells,

					fibroblasts, reviewed in [69,121]
Rhesus macaque	Peripheral neuropathy, fetal loss, subclinical [126,130,133,134,]	Pulmonary edema and erythema [131]	CNS: Meningitis, nuclear inclusions, vascular mineralization. Other: Interstitial pneumonia, lymphoid depletion, cytomegalic cells, hepatic necrosis, epididymitis[131]		

VZV, varicella zoster virus; SVV, simian varicella virus; HSV, herpes simplex virus

3.Duffy, M.R.; Chen, T.-H.; Hancock, W.T.; Powers, A.M.; Kool, J.L.; Lanciotti, R.S.; Pretrick, M.; Marfel, M.; Holzbauer, S.; Dubray, C.; et al. Zika Virus Outbreak on Yap Island, Federated States of Micronesia. *New England Journal of Medicine* **2009**, *360*, 2536-2543, doi:10.1056/NEJMoa0805715.

7 Miner, J.J.; Diamond, M.S. Zika Virus Pathogenesis and Tissue Tropism. *Cell host & microbe* **2017**, *21*, 134-142, doi:10.1016/j.chom.2017.01.004.

4. Diallo, D.; Sall, A.A.; Diagne, C.T.; Faye, O.; Faye, O.; Ba, Y.; Hanley, K.A.; Buenemann, M.; Weaver, S.C.; Diallo, M. Zika Virus Emergence in Mosquitoes in Southeastern Senegal, 2011. *PLOS ONE* **2014**, *9*, e109442, doi:10.1371/journal.pone.0109442.

5. Mansuy, J.M.; Dutertre, M.; Mengelle, C.; Fourcade, C.; Marchou, B.; Delobel, P.; Izopet, J.; Martin-Blondel, G. Zika virus: high infectious viral load in semen, a new sexually transmitted pathogen? *The Lancet Infectious Diseases* **2016**, *16*, 405, doi:https://doi.org/10.1016/S1473-3099(16)00138-9.

8. Komarasamy, T.V.; Adnan, N.A.A.; James, W.; Balasubramaniam, V. Zika Virus Neuropathogenesis: The Different Brain Cells, Host Factors and Mechanisms Involved. *Front Immunol* **2022**, *13*, 773191, doi:10.3389/fimmu.2022.773191.

24. Adams Waldorf, K.M.; Stencel-Baerenwald, J.E.; Kapur, R.P.; Studholme, C.; Boldenow, E.; Vornhagen, J.; Baldessari, A.; Dighe, M.K.; Thiel, J.; Merillat, S.; et al. Fetal brain lesions after subcutaneous inoculation of Zika virus in a pregnant nonhuman primate. *Nature medicine* **2016**, *22*, 1256-1259, doi:10.1038/nm.4193.

26. Robbiani, D.F.; Olsen, P.C.; Costa, F.; Wang, Q.; Oliveira, T.Y.; Nery, N., Jr.; Aromolaran, A.; do Rosário, M.S.; Sacramento, G.A.; Cruz, J.S.; et al. Risk of Zika microcephaly correlates with features of maternal antibodies. *J Exp Med* **2019**, *216*, 2302-2315, doi:10.1084/jem.20191061.

23. Adams Waldorf, K.M.; Nelson, B.R.; Stencel-Baerenwald, J.E.; Studholme, C.; Kapur, R.P.; Armistead, B.; Walker, C.L.; Merillat, S.; Vornhagen, J.; Tisoncik-Go, J.; et al. Congenital Zika virus infection as a silent pathology with loss of neurogenic output in the fetal brain. *Nature medicine* **2018**, *24*, 368-374, doi:10.1038/nm.4485.

25. Coffey, L.L.; Keesler, R.I.; Pesavento, P.A.; Woolard, K.; Singapuri, A.; Watanabe, J.; Cruzen, C.; Christe, K.L.; Usachenko, J.; Yee, J.; et al. Intraamniotic Zika virus inoculation of pregnant rhesus macaques produces fetal neurologic disease. *Nat Commun* **2018**, *9*, 2414-2414, doi:10.1038/s41467-018-04777-6.

16. Martinot, A.J.; Abbink, P.; Afacan, O.; Prohl, A.K.; Bronson, R.; Hecht, J.L.; Borducchi, E.N.; Larocca, R.A.; Peterson, R.L.; Rinaldi, W.; et al. Fetal Neuropathology in Zika Virus-Infected Pregnant Female Rhesus Monkeys. *Cell* **2018**, *173*, 1111-1122.e1110, doi:10.1016/j.cell.2018.03.019.

21. Steinbach, R.J.; Haese, N.N.; Smith, J.L.; Colgin, L.M.A.; MacAllister, R.P.; Greene, J.M.; Parkins, C.J.; Kempton, J.B.; Porsov, E.; Wang, X.; et al. A neonatal nonhuman primate model of gestational Zika virus infection with evidence of microencephaly, seizures and cardiomyopathy. *PloS one* **2020**, *15*, e0227676-e0227676, doi:10.1371/journal.pone.0227676.

14. Panganiban, A.T.; Blair, R.V.; Hattler, J.B.; Bohannon, D.G.; Bonaldo, M.C.; Schouest, B.; Maness, N.J.; Kim, W.-K. A Zika virus primary isolate induces neuroinflammation, compromises the blood-brain barrier and upregulates CXCL12 in adult macaques. *Brain Pathol* **2020**, *30*, 1017-1027, doi:10.1111/bpa.12873.

19. Block, L.N.; Aliota, M.T.; Friedrich, T.C.; Schotzko, M.L.; Mean, K.D.; Wiepz, G.J.; Golos, T.G.; Schmidt, J.K. Embryotoxic impact of Zika virus in a rhesus macaque in vitro implantation model†. *Biol Reprod* **2020**, *102*, 806-816, doi:10.1093/biolre/ioz236.
15. Hirsch, A.J.; Roberts, V.H.J.; Grigsby, P.L.; Haese, N.; Schabel, M.C.; Wang, X.; Lo, J.O.; Liu, Z.; Kroenke, C.D.; Smith, J.L.; et al. Zika virus infection in pregnant rhesus macaques causes placental dysfunction and immunopathology. *Nat Commun* **2018**, *9*, 263-263, doi:10.1038/s41467-017-02499-9.
20 Mohr, E.L.; Block, L.N.; Newman, C.M.; Stewart, L.M.; Koenig, M.; Semler, M.; Breitbach, M.E.; Teixeira, L.B.C.; Zeng, X.; Weiler, A.M.; et al. Ocular and uteroplacental pathology in a macaque pregnancy with congenital Zika virus infection. *PloS one* **2018**, *13*, e0190617-e0190617, doi:10.1371/journal.pone.0190617.

17. Gurung, S.; Reuter, N.; Preno, A.; Dubaut, J.; Nadeau, H.; Hyatt, K.; Singleton, K.; Martin, A.; Parks, W.T.; Papin, J.F.; et al. Zika virus infection at mid-gestation results in fetal cerebral cortical injury and fetal death in the olive baboon. *PLoS pathogens* **2019**, *15*, e1007507-e1007507, doi:10.1371/journal.ppat.1007507.
37. Sampson, B.A.; Ambrosi, C.; Charlot, A.; Reiber, K.; Veress, J.F.; Armbrustmacher, V. The pathology of human West Nile virus infection. *Human Pathology* **2000**, *31*, 527-531, doi:https://doi.org/10.1053/hp.2000.8047.
28. van Marle, G.; Antony, J.; Ostermann, H.; Dunham, C.; Hunt, T.; Halliday, W.; Maingat, F.; Urbanowski, M.D.; Hobman, T.; Peeling, J.; et al. West Nile Virus-Induced Neuroinflammation: Glial Infection and Capsid Protein-Mediated Neurovirulence. *Journal of Virology* **2007**, *81*, 10933-10949, doi:doi:10.1128/JVI.02422-06.
29. Samuel, M.A.; Wang, H.; Siddharthan, V.; Morrey, J.D.; Diamond, M.S. Axonal transport mediates West Nile virus entry into the central nervous system and induces acute flaccid paralysis. *Proc Natl Acad Sci U S A* **2007**, *104*, 17140-17145, doi:10.1073/pnas.0705837104.
31. Ratterree, M.S.; da Rosa, A.P.T.; Bohm, R.P.; Cogswell, F.B.; Phillippi, K.M.; Caillouet, K.; Schwanberger, S.; Shope, R.E.; Tesh, R.B. West Nile virus infection in nonhuman primate breeding colony, concurrent with human epidemic, southern Louisiana. **2003**.
33. Ratterree, M.S.; Gutierrez, R.A.; Travassos da Rosa, A.P.; Dille, B.J.; Beasley, D.W.; Bohm, R.P.; Desai, S.M.; Didier, P.J.; Bikenmeyer, L.G.; Dawson, G.J. Experimental infection of rhesus macaques with West Nile virus: level and duration of viremia and kinetics of the antibody response after infection. *The Journal of infectious diseases* **2004**, *189*, 669-676.
35. Verstrepen, B.E.; Fagrouch, Z.; van Heteren, M.; Buitendijk, H.; Haaksma, T.; Beenhakker, N.; Palù, G.; Richner, J.M.; Diamond, M.S.; Bogers, W.M.; et al. Experimental infection of rhesus macaques and common marmosets with a European strain of West Nile virus. *PLoS Negl Trop Dis* **2014**, *8*, e2797-e2797, doi:10.1371/journal.pntd.0002797.
36. Wertheimer, A.M.; Uhrlaub, J.L.; Hirsch, A.; Medigeshi, G.; Sprague, J.; Legasse, A.; Wilk, J.; Wiley, C.A.; Didier, P.; Tesh, R.B.; et al. Immune response to the West Nile virus in aged non-human primates. *PloS one* **2010**, *5*, e15514-e15514, doi:10.1371/journal.pone.0015514.
42. Maximova, O.A.; Speicher, J.M.; Skinner, J.R.; Murphy, B.R.; St Claire, M.C.; Ragland, D.R.; Herbert, R.L.; Pare, D.R.; Moore, R.M.; Pletnev, A.G. Assurance of neuroattenuation of a live vaccine against West Nile virus: a comprehensive study of neuropathogenesis after infection with chimeric WN/DEN4Δ30 vaccine in comparison to two parental viruses and a surrogate flavivirus reference vaccine. *Vaccine* **2014**, *32*, 3187-3197, doi:10.1016/j.vaccine.2014.04.002.
40. Pogodina, V.V.; Frolova, M.P.; Malenko, G.V.; Fokina, G.I.; Koreshkova, G.V.; Kiseleva, L.L.; Bochkova, N.G.; Ralph, N.M. Study on West Nile virus persistence in monkeys. *Archives of Virology* **1983**, *75*, 71-86, doi:10.1007/BF01314128.
38. Ølberg, R.-A.; Barker, I.K.; Crawshaw, G.J.; Bertelsen, M.F.; Drebot, M.A.; Andonova, M. West Nile virus encephalitis in a Barbary macaque (Macaca sylvanus). *Emerg Infect Dis* **2004**, *10*, 712.
46. Bastian, F.O.; Wende, R.D.; Singer, D.B.; Zeller, R.S. Eastern Equine Encephalomyelitis: Histopathologic and Ultrastructural Changes with Isolation of the Virus in a Human Case. *American Journal of Clinical Pathology* **1975**, *64*, 10-13, doi:10.1093/ajcp/64.1.10.
52. Lad, E.M.; Ong, S.S.; Proia, A.D. Ocular histopathology in Eastern equine encephalitis: A case report. *Am J Ophthalmol Case Rep* **2016**, *5*, 99-102, doi:10.1016/j.ajoc.2016.12.021.

66. Dahal, B.; Lin, S.-C.; Carey, B.D.; Jacobs, J.L.; Dinman, J.D.; van Hoek, M.L.; Adams, A.A.; Kehn-Hall, K. EGR1 upregulation following Venezuelan equine encephalitis virus infection is regulated by ERK and PERK pathways contributing to cell death. *Virology* **2020**, *539*, 121-128, doi:10.1016/j.virol.2019.10.016.

58. Reed, D.S.; Lackemeyer, M.G.; Garza, N.L.; Norris, S.; Gamble, S.; Sullivan, L.J.; Lind, C.M.; Raymond, J.L. Severe Encephalitis in Cynomolgus Macaques Exposed to Aerosolized Eastern Equine Encephalitis Virus. *The Journal of Infectious Diseases* **2007**, *196*, 441-450, doi:10.1086/519391.
57. Trefry, J.C.; Rossi, F.D.; Accardi, M.V.; Dorsey, B.L.; Sprague, T.R.; Wollen-Roberts, S.E.; Shamblin, J.D.; Kimmel, A.E.; Glass, P.J.; Miller, L.J.; et al. The utilization of advance telemetry to investigate critical physiological parameters including electroencephalography in cynomolgus macaques following aerosol challenge with eastern equine encephalitis virus. *PLoS Negl Trop Dis* **2021**, *15*, e0009424-e0009424, doi:10.1371/journal.pntd.0009424.
43. Weaver, S.C.; Winegar, R.; Manger, I.D.; Forrester, N.L. Alphaviruses: population genetics and determinants of emergence. *Antiviral research* **2012**, *94*, 242-257, doi:10.1016/j.antiviral.2012.04.002.
56. Albe, J.R.; Ma, H.; Gilliland, T.H.; McMillen, C.M.; Gardner, C.L.; Boyles, D.A.; Cottle, E.L.; Dunn, M.D.; Lundy, J.D.; O'Malley, K.J.; et al. Physiological and immunological changes in the brain associated with lethal eastern equine encephalitis virus in macaques. *PLoS pathogens* **2021**, *17*, e1009308-e1009308, doi:10.1371/journal.ppat.1009308.
44. Smith, D.R.; Schmaljohn, C.S.; Badger, C.; Ostrowski, K.; Zeng, X.; Grimes, S.D.; Rayner, J.O. Comparative pathology study of Venezuelan, eastern, and western equine encephalitis viruses in non-human primates. *Antiviral Research* **2020**, *182*, 104875, doi:https://doi.org/10.1016/j.antiviral.2020.104875.
59. Porter, A.I.; Erwin-Cohen, R.A.; Twenhafel, N.; Chance, T.; Yee, S.B.; Kern, S.J.; Norwood, D.; Hartman, L.J.; Parker, M.D.; Glass, P.J.; et al. Characterization and pathogenesis of aerosolized eastern equine encephalitis in the common marmoset (Callithrix jacchus). *Virol J* **2017**, *14*, 25-25, doi:10.1186/s12985-017-0687-7.

53. Cain, M.D.; Salimi, H.; Gong, Y.; Yang, L.; Hamilton, S.L.; Heffernan, J.R.; Hou, J.; Miller, M.J.; Klein, R.S. Virus entry and replication in the brain precedes blood-brain barrier disruption during intranasal alphavirus infection. *Journal of neuroimmunology* **2017**, *308*, 118-130, doi:10.1016/j.jneuroim.2017.04.008.

61. Burke, C.W.; Froude, J.W.; Rossi, F.; White, C.E.; Moyer, C.L.; Ennis, J.; Pitt, M.L.; Streatfield, S.; Jones, R.M.; Musiychuk, K.; et al. Therapeutic monoclonal antibody treatment protects nonhuman primates from severe Venezuelan equine encephalitis virus disease after aerosol exposure. *PLoS pathogens* **2019**, *15*, e1008157-e1008157, doi:10.1371/journal.ppat.1008157.
62. Ma, H.; Lundy, J.D.; Cottle, E.L.; O'Malley, K.J.; Trichel, A.M.; Klimstra, W.B.; Hartman, A.L.; Reed, D.S.; Teichert, T. Applications of minimally invasive multimodal telemetry for continuous monitoring of brain function and intracranial pressure in macaques with acute viral encephalitis. *PloS one* **2020**, *15*, e0232381-e0232381, doi:10.1371/journal.pone.0232381.
63. Ma, H.; Lundy, J.D.; O'Malley, K.J.; Klimstra, W.B.; Hartman, A.L.; Reed, D.S. Electrocardiography Abnormalities in Macaques after Infection with Encephalitic Alphaviruses. *Pathogens* **2019**, *8*, 240, doi:10.3390/pathogens8040240.
60. Reed, D.S.; Lind, C.M.; Sullivan, L.J.; Pratt, W.D.; Parker, M.D. Aerosol Infection of Cynomolgus Macaques with Enzootic Strains of Venezuelan Equine Encephalitis Viruses. *The Journal of Infectious Diseases* **2004**, *189*, 1013-1017, doi:10.1086/382281.
64. Rossi, S.L.; Russell-Lodrigue, K.E.; Killeen, S.Z.; Wang, E.; Leal, G.; Bergren, N.A.; Vinet-Oliphant, H.; Weaver, S.C.; Roy, C.J. IRES-Containing VEEV Vaccine Protects Cynomolgus Macaques from IE Venezuelan Equine Encephalitis Virus Aerosol Challenge. *PLoS Negl Trop Dis* **2015**, *9*, e0003797-e0003797, doi:10.1371/journal.pntd.0003797.
65
. Monath, T.P.; Calisher, C.H.; Davis, M.; Bowen, G.S.; White, J. Experimental Studies of Rhesus Monkeys Infected with Epizootic and Enzootic Subtypes of Venezuelan Equine Encephalitis Virus. *The Journal of Infectious Diseases* **1974**, *129*, 194-200, doi:10.1093/infdis/129.2.194.

48. Bruyn, H.B.; Lennette, E.H. Western equine encephalitis in infants; a report on three cases with sequelae. *Calif Med* **1953**, *79*, 362-366.
68. Reed, D.S.; Larsen, T.; Sullivan, L.J.; Lind, C.M.; Lackemeyer, M.G.; Pratt, W.D.; Parker, M.D. Aerosol Exposure to Western Equine Encephalitis Virus Causes Fever and Encephalitis in Cynomolgus Macaques. *The Journal of Infectious Diseases* **2005**, *192*, 1173-1182, doi:10.1086/444397.
80. Suzuki, T.; Tetsuka, S.; Ogawa, T.; Hashimoto, R.; Okada, S.; Kato, H. An Autopsy Case of Varicella Zoster Virus Encephalitis with Multiple Brain Lesions. *Intern Med* **2020**, *59*, 1643-1647, doi:10.2169/internalmedicine.3417-19.
81. Bakradze, E.; Kirchoff, K.F.; Antoniello, D.; Springer, M.V.; Mabie, P.C.; Esenwa, C.C.; Labovitz, D.L.; Liberman, A.L. Varicella Zoster Virus Vasculitis and Adult Cerebrovascular Disease. *Neurohospitalist* **2019**, *9*, 203-208, doi:10.1177/1941874419845732.
94. Bubak, A.N.; Traina-Dorge, V.; Como, C.N.; Feia, B.; Pearce, C.M.; Doyle-Meyers, L.; Das, A.; Looper, J.; Mahalingam, R.; Nagel, M.A. Elevated serum substance P during simian varicella virus infection in rhesus macaques: implications for chronic inflammation and adverse cerebrovascular events. *Journal of neurovirology* **2020**, *26*, 945-951, doi:10.1007/s13365-020-00907-3.

83. Traina-Dorge, V.; Palmer, B.E.; Coleman, C.; Hunter, M.; Frieman, A.; Gilmore, A.; Altrock, K.; Doyle-Meyers, L.; Nagel, M.A.; Mahalingam, R. Reactivation of Simian Varicella Virus in Rhesus Macaques after CD4 T Cell Depletion. *Journal of virology* **2019**, *93*, e01375-01318, doi:10.1128/JVI.01375-18.

97. Tognarelli, E.I.; Palomino, T.F.; Corrales, N.; Bueno, S.M.; Kalergis, A.M.; González, P.A. Herpes Simplex Virus Evasion of Early Host Antiviral Responses. *Front Cell Infect Microbiol* **2019**, *9*, 127-127, doi:10.3389/fcimb.2019.00127.
69. Connolly, S.A.; Jardetzky, T.S.; Longnecker, R. The structural basis of herpesvirus entry. *Nat Rev Microbiol* **2021**, *19*, 110-121, doi:10.1038/s41579-020-00448-w.
113. Lo, M.; Zhu, J.; Hansen, S.G.; Carroll, T.; Farr Zuend, C.; Nöel-Romas, L.; Ma, Z.-M.; Fritts, L.; Huang, M.-L.; Sun, S.; et al. Acute Infection and Subsequent Subclinical Reactivation of Herpes Simplex Virus 2 after Vaginal Inoculation of Rhesus Macaques. *Journal of virology* **2019**, *93*, e01574-01518, doi:10.1128/JVI.01574-18.
109. Fan, S.; Cai, H.; Xu, X.; Feng, M.; Wang, L.; Liao, Y.; Zhang, Y.; He, Z.; Yang, F.; Yu, W.; et al. The Characteristics of Herpes Simplex Virus Type 1 Infection in Rhesus Macaques and the Associated Pathological Features. *Viruses* **2017**, *9*, 26, doi:10.3390/v9020026.
110. Aravantinou, M.; Frank, I.; Arrode-Bruses, G.; Szpara, M.; Grasperge, B.; Blanchard, J.; Gettie, A.; Derby, N.; Martinelli, E. A model of genital herpes simplex virus Type 1 infection in Rhesus Macaques. *J Med Primatol* **2017**, *46*, 121-128, doi:10.1111/jmp.12293.

118. Costa, É.A.; Luppi, M.M.; de Campos Cordeiro Malta, M.; Luiz, A.P.M.F.; de Araujo, M.R.; Coelho, F.M.; Fonseca, F.G.d.; Ecco, R.; Resende, M. Outbreak of Human Herpesvirus Type 1 Infection in Nonhuman Primates (Callithrix penincillata). *Journal of Wildlife Diseases* **2011**, *47*, 690-693, doi:10.7589/0090-3558-47.3.690.
115. Edwards, E.E.; Birch, S.M.; Hoppes, S.M.; Keating, M.K.; Stoica, G. Pathology in Practice. *Journal of the American Veterinary Medical Association* **2018**, *253*, 423-426, doi:10.2460/javma.253.4.423.
119. Imura, K.; Chambers, J.K.; Uchida, K.; Nomura, S.; Suzuki, S.; Nakayama, H.; Miwa, Y. Herpes simplex virus type 1 infection in two pet marmosets in Japan. *J Vet Med Sci* **2014**, *76*, 1667-1670, doi:10.1292/jvms.14-0374.
120. Longa, C.S.; Bruno, S.F.; Pires, A.R.; Romijn, P.C.; Kimura, L.S.; Costa, C.H.C. Human herpesvirus 1 in wild marmosets, Brazil, 2008. *Emerg Infect Dis* **2011**, *17*, 1308-1310, doi:10.3201/eid1707.100333.
122. Maltezou, P.-G.; Kourlaba, G.; Kourkouni, E.; Luck, S.; Blázquez-Gamero, D.; Ville, Y.; Lilleri, D.; Dimopoulou, D.; Karalexi, M.; Papaevangelou, V. Maternal type of CMV infection and sequelae in infants with congenital CMV: Systematic review and meta-analysis. *Journal of Clinical Virology* **2020**, *129*, 104518, doi:https://doi.org/10.1016/j.jcv.2020.104518.
123. Fowler, K.B.; Boppana, S.B. Congenital cytomegalovirus infection. *Seminars in Perinatology* **2018**, *42*, 149-154, doi:https://doi.org/10.1053/j.semperi.2018.02.002.
121. Griffiths, P.; Reeves, M. Pathogenesis of human cytomegalovirus in the immunocompromised host. *Nat Rev Microbiol* **2021**, *19*, 759-773, doi:10.1038/s41579-021-00582-z.

134 Fan, Q.; Nelson, C.S.; Bialas, K.M.; Chiuppesi, F.; Amos, J.; Gurley, T.C.; Marshall, D.J.; Eudailey, J.; Heimsath, H.; Himes, J.; et al. Plasmablast Response to Primary Rhesus Cytomegalovirus (CMV) Infection in a Monkey Model of Congenital CMV Transmission. *Clin Vaccine Immunol* **2017**, *24*, e00510-00516, doi:10.1128/CVI.00510-16.
133 Bialas, K.M.; Tanaka, T.; Tran, D.; Varner, V.; Cisneros De La Rosa, E.; Chiuppesi, F.; Wussow, F.; Kattenhorn, L.; Macri, S.; Kunz, E.L.; et al. Maternal CD4+ T cells protect against severe congenital cytomegalovirus disease in a novel nonhuman primate model of placental cytomegalovirus transmission. *Proc Natl Acad Sci U S A* **2015**, *112*, 13645-13650, doi:10.1073/pnas.1511526112.
130. Lockridge, K.M.; Sequar, G.; Zhou, S.S.; Yue, Y.; Mandell, C.P.; Barry, P.A. Pathogenesis of experimental rhesus cytomegalovirus infection. *Journal of virology* **1999**, *73*, 9576-9583, doi:10.1128/JVI.73.11.9576-9583.1999.
126Marsh, A.K.; Ambagala, A.P.; Perciani, C.T.; Russell, J.N.H.; Chan, J.K.; Janes, M.; Antony, J.M.; Pilon, R.; Sandstrom, P.; Willer, D.O.; et al. Examining the species-specificity of rhesus macaque cytomegalovirus (RhCMV) in cynomolgus macaques. *PloS one* **2015**, *10*, e0121339-e0121339, doi:10.1371/journal.pone.0121339.
131Baskin, G.B. Disseminated cytomegalovirus infection in immunodeficient rhesus monkeys. *Am J Pathol* **1987**, *129*, 345-352.
